# Development of Clindamycin-Releasing Polyvinyl Alcohol Hydrogel with Self-Healing Property for the Effective Treatment of Biofilm-Infected Wounds

**DOI:** 10.3390/gels10070482

**Published:** 2024-07-19

**Authors:** Nur Alifah, Juliana Palungan, Kadek Ardayanti, Muneeb Ullah, Andi Nokhaidah Nurkhasanah, Apon Zaenal Mustopa, Subehan Lallo, Rina Agustina, Jin-Wook Yoo, Nurhasni Hasan

**Affiliations:** 1 Faculty of Pharmacy, Hasanuddin University, Jl. Perintis Kemerdekaan KM 10, Makassar 90245, Indonesia; nurr.aalifah@gmail.com (N.A.); palunganjuliana@gmail.com (J.P.); kadekardayanti@gmail.com (K.A.); andi.nokhaidah@gmail.com (A.N.N.); subehan@unhas.ac.id (S.L.); rinaagustina@unhas.ac.id (R.A.); 2 College of Pharmacy, Pusan National University, Busan 46241, Republic of Korea; munibdawar72@gmail.com (M.U.); jinwook@pusan.ac.kr (J.-W.Y.); 3 Research Center for Genetic Engineering, National Research and Innovation Agency (BRIN), Bogor 16911, Indonesia; azae001@brin.go.id

**Keywords:** bacterial biofilm infection, clindamycin, polyvinyl alcohol, borax, self-healing hydrogel

## Abstract

Self-healing hydrogels have good mechanical strength, can endure greater external force, and have the ability to heal independently, resulting in a strong bond between the wound and the material. Bacterial biofilm infections are life-threatening. Clindamycin (Cly) can be produced in the form of a self-healing hydrogel preparation. It is noteworthy that the antibacterial self-healing hydrogels show great promise as a wound dressing for bacterial biofilm infection. In this study, we developed a polyvinyl alcohol/borax (PVA/B) self-healing hydrogel wound dressing that releases Cly. Four ratios of PVA, B, and Cly were used to make self-healing hydrogels: F1 (4%:0.8%:1%), F2 (4%:1.2%:1%), F3 (1.6%:1%), and F4 (4%:1.6%:0). The results showed that F4 had the best physicochemical properties, including a self-healing duration of 11.81 ± 0.34 min, swelling ratio of 85.99 ± 0.12%, pH value of 7.63 ± 0.32, and drug loading of 98.34 ± 11.47%. The B–O–C cross-linking between PVA and borax caused self-healing, according to FTIR spectra. The F4 formula had a more equal pore structure in the SEM image. The PVA/B-Cly self-healing hydrogel remained stable at 6 ± 2 °C for 28 days throughout the stability test. The Korsmeyer–Peppas model released Cly by Fickian diffusion. In biofilm-infected mouse wounds, PVA/B-Cly enhanced wound healing and re-epithelialization. Our results indicate that the PVA/B-Cly produced in this work has reliable physicochemical properties for biofilm-infected wound therapy.

## 1. Introduction

A wound refers to an injury or disturbance to the natural structure of the skin, which hinders its physiological function [1]. Bacterial infections in wounds impair and delay the healing processes. Methicillin-resistant Staphylococcus aureus (MRSA) is the predominant bacteria responsible for wound infections, which pose a significant challenge in treatment due to their resistance to multiple antibiotics including penicillin, cephalosporin, teicoplanin, and vancomycin [2,3]. Bacteria can exacerbate the situation by forming a biofilm on the wound, which serves as a defensive mechanism against antibiotics. A biofilm is formed when a collection of microbial cells, which are attached to a surface, are enclosed within a matrix called an extracellular polymeric material [4,5]. Clindamycin (Cly) is still an effective second-line treatment for bacterial infections in skin wounds and soft tissue, even in the presence of antibiotic-resistant bacteria like MRSA [2,6].

Clindamycin, a semi-synthetic antibiotic originating from lincomycin, has received approval from the Food and Drug Administration (FDA) for the treatment of MRSA bacterial infections [7,8]. This antibiotic functions by attaching itself to the 50S component of the bacterial ribosome, thereby impeding the process of protein synthesis. Nevertheless, the oral administration of Cly can lead to first-pass metabolism in the liver, hence impeding the oral utilization of Cly [7]. It exhibits excellent tissue penetration, making it highly effective for the topical treatment of severe wounds, including skin and soft tissue infections [9,10].

An excellent approach to enhancing the efficacy of Cly in the treatment of bacterial infections in wounds is to formulate it as a wound dressing. This dressing can provide protection and expedite the wound-healing process. Cly can be found in a variety of wound dressings, including patches, films, and hydrogels. Particular dosage forms can cause pain during wound dressing changes and cannot be utilized in particular regions like the elbow, wrist, or foot due to the possibility of breaking or cracking the wound dressing [7,11]. Hence, a self-healing hydrogel was created, capable of restoring its original form following mechanical injury. This property improves the durability of wound dressings and allows them to be applied to specific areas such as knees, elbows, wrists, and feet [12]. 

Hydrogels possess a self-healing mechanism through the reversible nature of their cross-link structure, which consists of dynamic cross-links and dynamic non-covalent connections. These components have the ability to spontaneously regenerate once the material has been damaged [12]. Polyvinyl alcohol (PVA) is a synthetic polymer commonly utilized in the production of self-healing hydrogels due to its water-loving properties, compatibility with living organisms, lack of toxicity, and ability to break down naturally over time [13,14]. Furthermore, PVA possesses a substantial number of hydroxyl groups; this allows it to return to its former shape on its own by forming hydrogen bonds between molecular chains [15]. Nevertheless, hydrogels containing only PVA exhibit challenges in terms of malleability and mechanical strength, necessitating the incorporation of a cross-linking agent [16].

Sodium tetraborate, or borax (B), is a cross-linking agent that is commonly employed in water-soluble polymers due to its low toxicity and high solubility in water. When borax is exposed to water, it undergoes hydrolysis and transforms into boric acid and borate ions [17,18]. Borax acts as a cross-linker, causing the PVA chains to bond together. This leads to the solidification of PVA and entrapment of water molecules, culminating in the formation of a slimy mass. The formation of cross-links between PVA and borax occurs when two PVA diol units react with one borate ion from borax, resulting in a di-diol complexation reaction. This reaction leads to the creation of a self-healing hydrogel that exhibits a substantial increase in flexibility [19,20].

**Scheme 1:**
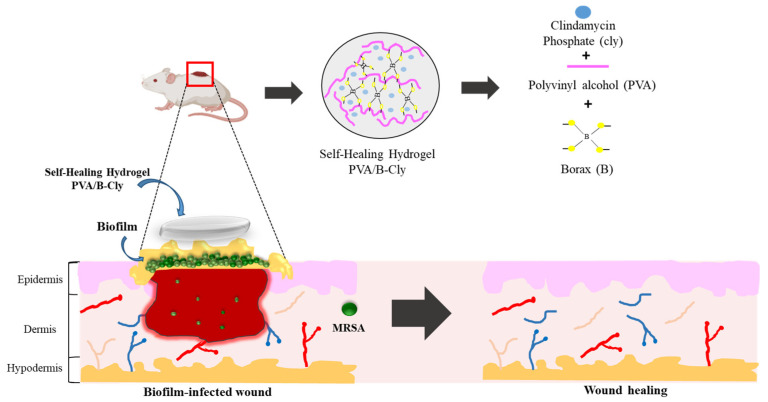
Scheme of the cross-linking reaction of polyvinyl alcohol (PVA) and borax (B) to form the PVA/B-Cly self-healing hydrogel for the treatment of biofilm-infected wounds.

In the present work, we developed a Cly-releasing PVA/B self-healing hydrogel for the effective treatment of biofilm-infected wounds (Scheme 1). The self-healing hydrogels are formed by cross-linking PVA and borax at various concentration ratios. We investigated hydrogels for in vitro drug release after assessing their wound-dressing physicochemical properties. Finally, we assessed the in vivo wound-healing activity using an ICR mouse model of biofilm-infected wounds.

## 2. Results and Discussion

### 2.1. Preparation of the PVA/B and PVA/B-Cly Self-Healing Hydrogel

**Figure 1:**
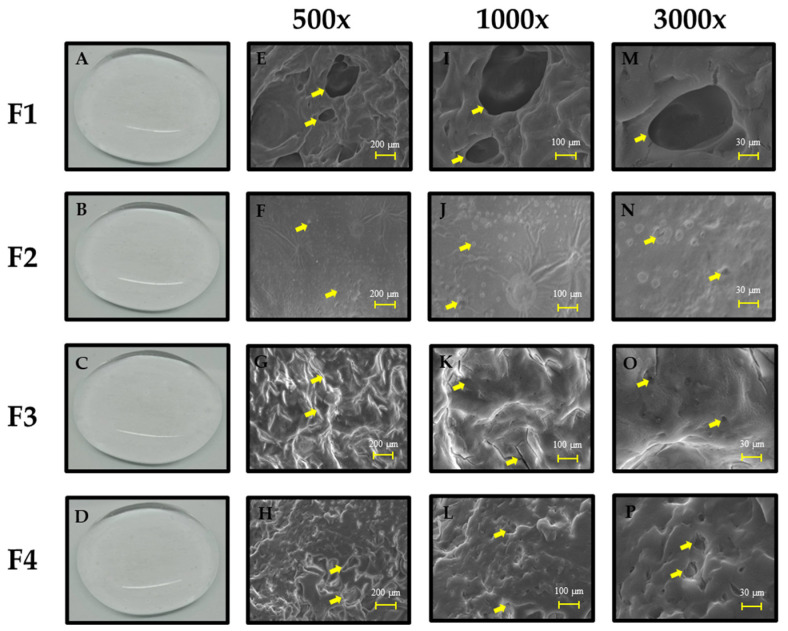
The macroscopic and microscopic images of PVA/B and PVA/B-Cly self-healing hydrogels with three different ratios of PVA, borax, and clindamycin. Macroscopic image of self-healing hydrogel (**A**) F1 (4%:1.6%:0%), (**B**) F2 (4%:0.8%:1%), (**C**) F3 (4%:1.2%:1%), and (**D**) F4 (4%:1.6%:1%). Scanning electron microscope (SEM) images of F1, F2, F3, and F4 at 500× (**E**–**H**), 1000× (**I**–**L**), and 3000× (**M**–**P**). Yellow arrows represent pores of the self-healing hydrogel.

Self-healing hydrogels have emerged as a highly intriguing synthetic biomaterial in recent decades. Its primary characteristic is its ability to restore its original qualities and structure following mechanical injury [21]. A self-healing hydrogel that releases Cly, comprising PVA as a polymer, borax as a cross-linking agent, and Cly as an active pharmaceutical ingredient, was produced, described, and evaluated. PVA is a polymer that can dissolve in water, is biocompatible, is non-toxic, and can be easily cross-linked [22]. Nevertheless, the utilization of a solitary PVA exhibits inadequate mechanical characteristics and is not readily moldable. In order to tie the PVA chains together, the inclusion of a cross-linking agent is necessary [16]. The selection of borax as a cross-linking agent was based on its ability to create reversible cross-links that can naturally regenerate once the self-healing hydrogel is damaged [20]. The self-healing hydrogel was successfully developed with different concentrations of PVA, borax, and Cly (Table 1), namely F1 (4%:1.6%:0%), F2 (4%:0.8%:1%), F3 (4%:1.2%:1%), and F4 (4%:1.6%:1%). F1 was the formula without Cly; therefore, it hereafter refers to the PVA/B hydrogel. Meanwhile, F2–F4 were formulas containing Cly and further refer to PVA/B-Cly hydrogels. The self-healing hydrogel obtained had a smooth surface that was evenly distributed. The physical appearances of PVA/B and PVA/B-Cly self-healing hydrogels is shown in Figure 1A–D. The self-healing hydrogels were round, transparent, and contained a significant amount of water, which reveal their hydrogel state.

### 2.2. Physicochemical Characterization of the Self-Healing Hydrogel

#### 2.2.1. Morphological Analysis of Self-Healing Hydrogel PVA/B and PVA/B-Cly

The freeze-dried preparations of PVA/B and PVA/B-Cly self-healing hydrogels revealed a distinct three-dimensional network morphology, characterized by a porous network structure. This is seen in Figure 1E–P. Morphological observations were carried out to determine the effect of borax concentration as a cross-linking agent on the self-healing hydrogel [22]. Formula F4 (4%:1.6%) exhibits a greater and more uniform pore size in comparison with F2 (4%:0.8%) and F3 (4%:1.2%). Higher concentrations of borax as a cross-linking agent led to a greater degree of cross-linking, resulting in a denser and more compact structure. This, in turn, leads to the formation of more uniform and regular pores [23]. Meanwhile, F1, in the absence of Cly, exhibited bigger and more irregular pores compared with F4, despite having the same concentration as PVA/borax. This phenomenon is likely attributed to the non-uniform formation of cross-links resulting from the uneven distribution of the cross-linking agent within the self-healing hydrogel. Consequently, the cross-links tend to accumulate in specific regions, leading to the formation of bigger pores in other areas [14].

#### 2.2.2. FTIR Characterization of Self-Healing Hydrogel

**Figure 2:**
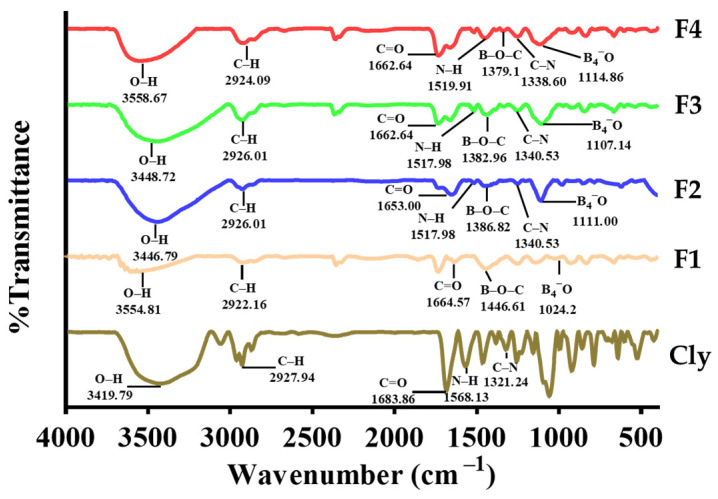
Fourier transform infrared spectroscopy (FTIR) spectra of clindamycin phosphate and self-healing hydrogels PVA/B (F1) and PVA/B-Cly (F2, F3, and F4).

An FTIR analysis was used to detect the functional groups present in the self-healing hydrogel (Figure 2). In addition, the objective was to detect the presence of the B–O–C group, which serves as a cross-link between PVA and borax. The wave numbers 1446.61–1379.1 cm^−1^ showed the presence of the B–O–C functional group, while the wave numbers 1114.86–1024.2 cm^−1^ corresponded to the remaining borate ions derived from borax. B–O–C cross-links were created by the interaction of two diol units from PVA and one borate ion from borax [20]. The B–O–C group reached its maximum strength in F4 and F1, where the borax concentration was highest at 1.6% in contrast to F2 and F3. The concentration of borax affected the intensity of the B–O–C bonds in PVA/B and PVA/B-Cly self-healing hydrogels. According to Huang et al. (2017) [23], increasing the concentration of borax leads to a greater number of cross-links being generated, resulting in a higher intensity of the cross-links. In addition, the C–N and N–H functional groups are classified as Cly functional groups and can be observed at wave numbers ranging from 1340.53 to 1338.6 cm^−1^ and from 1519.91 to 1517.98 cm^−1^ [24]. These functional groups were present in F2, F3, and F4. This demonstrated that the process of combining and creating connections between PVA and borax does not have any impact on or does not alter the composition of Cly. 

#### 2.2.3. Self-Healing Behavior of PVA/B and PVA/B-Cly

**Figure 3:**
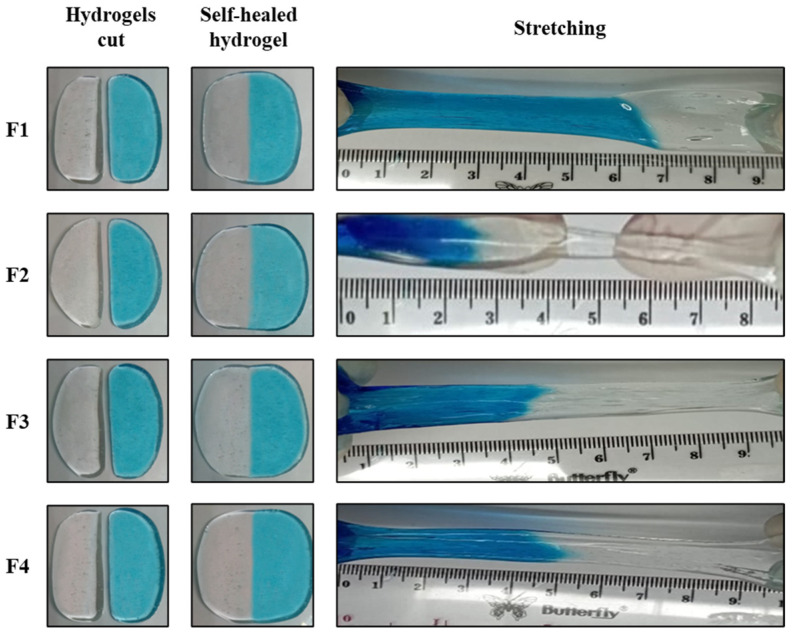
Self-healing behavior of PVA/B (F1) and PVA/B-Cly (F2, F3, F4). The self-healing hydrogel can be extended up to 100% of its initial length; it was sliced in half, and it was shown that self-healing behavior results from the two separate parts being brought into contact for one hour without any outside stimulus.

Figure 3 depicts the recuperative properties of PVA/B and PVA/B-Cly self-healing hydrogels. The primary requirement for a self-healing hydrogel is the ability to regain its original shape following mechanical damage, known as its self-healing feature. The self-healing capability, when implemented, can effectively prevent damage or cracks in the preparation, hence optimizing its potential to protect and treat bacterial infections in wounds [12]. The self-healing hydrogel, consisting of two components, has the ability to seamlessly rejoin after being cut and can also be stretched without developing surface fissures. The experiment involved partitioning the self-healing hydrogel into two segments, with one segment being treated with methylene blue dye. The preparation was again positioned in the identical location, and the duration required for the self-healing hydrogel to reestablish itself at room temperature without any external stimulation was monitored. Table 2 displays the reintegration time, indicating that F2 had a faster self-healing time of 6.96 min compared with F3 and F4. The self-healing durations of F3 and F4 were 9.18 min and 11.81 min, respectively. The self-healing time of blank self-healing hydrogel (F1) was 14.45 min, which was longer than F4 with the same concentration of polymer and cross-linking agent. The self-healing time varied depending on the concentration of borax used as a cross-linking agent. The reason for this is because when the concentration of borax increases, there is a tendency for more cross-links to form, leading to a denser and more compact structure. Consequently, more time is required for the preparation to reintegrate [23,25]. According to Wang et al. (2021), self-healing ability occurs at 2, 15, 30, and 60 min. The self-healing will be more impeccable the longer the contact time between self-healing hydrogel pieces. As the contact duration between individual pieces of self-healing hydrogel increases, so will their capacity to resist motion or friction [14].

#### 2.2.4. pH Characterization of Self-Healing Hydrogel

The pH values of PVA/B (F1) and PVA/B-Cly (F2, F3, and F4) self-healing hydrogels are shown in Table 2. The pH values for all formulas were close to neutral, with values measuring 7.36, 7.56, 7.65, and 7.63 for F1, F2, F3, and F4, respectively. The statistical results showed that there was no significant difference between the pH values of blank self-healing hydrogel and Cly-releasing self-healing hydrogel (*p* > 0.05). The pH of healthy and normal skin often falls within the slightly acidic range of 4.0 to 6.0 [26]. When a wound occurs on the skin, the pH level will shift from a neutral state to an alkaline state. Open wounds often have a pH level that is neither acidic nor alkaline, ranging from 6.5 to 8.5. On the other hand, chronic wounds tend to have a pH level ranging from 7.2 to 8.9 [27]. As the wound goes through the healing stages, its pH will transition from neutral to acidic. This shift in pH is a significant factor in predicting the outcome of wound healing, as indicated by Goswami et al. (2023). Lowering the alkaline pH level in the wound area will hinder the function of protease enzymes, which can degrade newly created tissue during the wound-healing process, hence ensuring the uninterrupted progression of wound healing [28,29]. Therefore, the PVA/B-Cly self-healing hydrogel is suitable for use as a wound dressing due to its almost neutral pH value, which ensures it does not exacerbate or impede the wound-healing process.

#### 2.2.5. Drug Content

The Cly content in PVA/B-Cly self-healing hydrogel from three different formulations, namely F2, F3, and F4, is shown in Table 2. The drug contents in F2, F3, and F4 were 98.72%, 97.77%, and 98.34%, respectively.

#### 2.2.6. Swelling Ratio of Self-Healing Hydrogel

**Figure 4:**
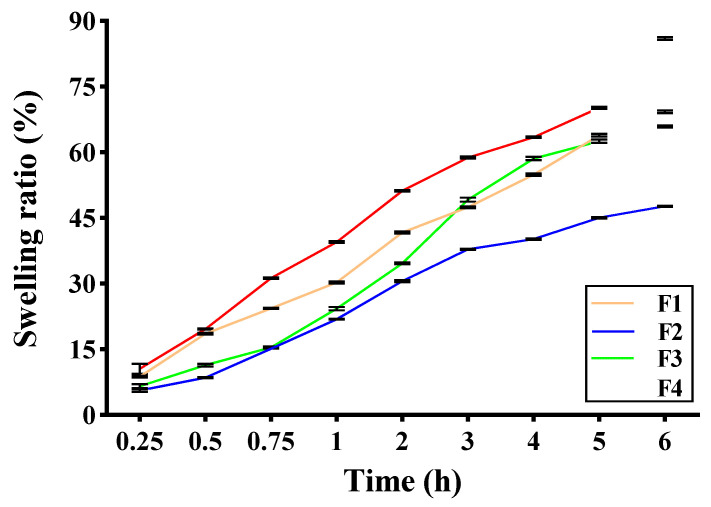
Swelling properties of PVA/B (F1) and PVA/B-Cly (F2, F3, F4) self-healing hydrogels. The data, with *n* = 3, are displayed as the mean ± standard deviation.

Hydrogels possess the capacity to swell and retain a substantial quantity of water. When used in a therapeutic setting, a self-healing hydrogel soaked in PBS (pH 7.4) has the ability to expand, allowing it to collect exudate and provide a moist environment for the wound. The self-healing hydrogels PVA/B and PVA/B-Cly rapidly absorbed PBS, reaching around 85.99% of their original weight in the F4 formulation. They achieved maximal absorption after 6 h and caused a change in the form of a self-healing hydrogel. The swelling ratios of F1, F2, and F3 were 69.19%, 47.67%, and 65.91%, respectively (Figure 4). Self-healing hydrogels possess the advantageous characteristic of expanding when they come into contact with solvents that are thermodynamically suitable [30]. An optimal degree of swelling facilitates the absorption of wound exudate and the preservation of moisture in the wound region, hence enhancing the wound-healing process [31]. Formula F4 has the greatest swelling ratio when compared with Formulas 1, 2, and 3. Higher concentrations of borax as a cross-linking agent result in the formation of larger and more irregular pore sizes in hydrogels. This allows them to have a greater capacity for water absorption compared with self-healing hydrogels, which have smaller pore sizes [23,31]. This is due to the larger void volume, which allows for greater water retention capacity compared with a self-healing hydrogel with a smaller pore size [20]. The self-healing hydrogel (F1) without Cly exhibited a reduced swelling ratio compared with F4, despite both having the same PVA/B content. The extensive cross-linking in F1 results in the formation of a hydrogel with a more condensed and tightly packed structure compared with F4. As a consequence, F1 exhibits a diminished capacity for water absorption [14,23].

#### 2.2.7. Stability Study

**Figure 5:**
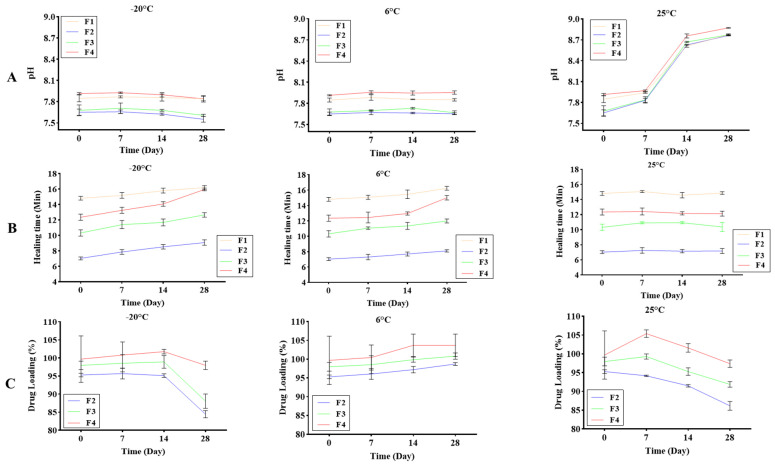
The stability of self-healing hydrogels evaluated at various temperatures: (**A**) pH, (**B**) healing time, and (**C**) drug loading.

The stability of the self-healing hydrogel was assessed under three distinct temperature storage conditions, freezing temperature (−20 °C), cold temperature (6 °C), and room temperature (25 °C), over a period of 28 days. There was no alteration in the shape, color, and odor of the PVA/B and PVA/B-Cly self-healing hydrogel during the 28-day storage period. Nevertheless, at a temperature of 25 °C, the self-healing hydrogel exhibited mold formation, although no data were provided to support this observation. The pH value of self-healing PVA/B and PVA/B-Cly self-healing hydrogels remained constant while stored at 6 °C (Figure 5). Concurrently, the pH value rose at a storage temperature of 25 °C and decreased at a temperature of −20 °C. The self-healing duration following a 28-day storage period remained constant when held at a temperature of 25 °C. Nevertheless, notable alterations occurred at storage temperatures of 6 °C and −20 °C. At a temperature of 6 °C, there was no reduction in the Cly content, suggesting that the Cly content in the self-healing hydrogel remained constant prior to storage. After 28 days of storage, the breakdown rate of Cly showed a considerable decrease at the temperatures of −20 °C and 25 °C.

Stability testing was conducted at three distinct temperatures (−20, 6, and 25 °C) in order to assess the stability of the self-healing hydrogel. The evaluation included the analysis of organoleptic properties, self-healing time, pH, and drug content. Changes in the drug and therapeutic characteristics of each drug component, including both active substances and excipients, will lead to instability [32]. Inadequate temperature control, elevated humidity, or light exposure can lead to fast disintegration by generating several breakdown byproducts [33]. Over a period of 28 days, the PVA/B and PVA/B-Cly self-healing hydrogels did not undergo any alterations in their shape, color, or odor when stored at different temperatures. However, when stored at a temperature of 25 °C, microbial contamination occurred, as evidenced by the presence of fungal growth around the preparation on the 14th day. Water-based formulations provide an ideal environment for microbial growth due to their ability to support active development at optimal temperatures, such as 20 °C [34]. pH stability testing revealed no substantial alterations when stored at a temperature of 6 °C. Nevertheless, the pH level rose at a storage temperature of 25 °C and decreased to −20 °C. The pH stability is a crucial factor as it directly impacts the effectiveness of wound healing [35]. The duration of self-healing is influenced by the temperature at which the storage occurs. The duration of reintegration will be extended when the storage temperature of the self-healing hydrogel decreases. At a temperature of 25 °C, there was no alteration in the time it took for self-healing to occur. However, at temperatures of 6 °C and −20 °C, there was a highly notable alteration in the time it took for self-healing to occur after storage. The Cly content in the PVA/B-Cly self-healing hydrogel remained constant at a temperature of 6 °C and satisfied the criteria for optimal drug content in the formulation, specifically within the range of 95.0–105.0%. The Cly concentrations remained consistent at 84% in F2, 88% in F3, and 97% in F4 while stored at −20 °C. When held at a temperature of 25 °C, the percentage of Cly remaining was 86% in F2, 91% in F3, and 97% in F4. Therefore, a storage temperature of 6 °C is optimal for PVA/B-Cly self-healing hydrogel as it ensures minimal alterations in form, color, odor, pH, and drug content while also promoting effective self-healing.

#### 2.2.8. In Vitro Drug Release

**Figure 6:**
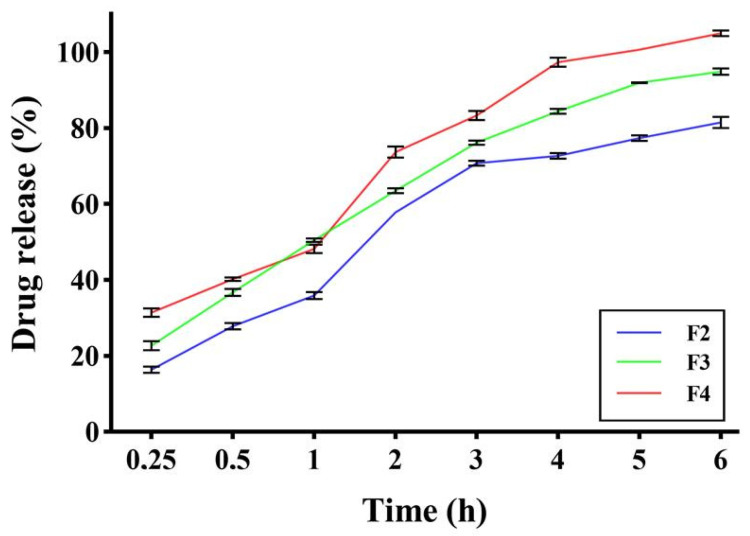
The in vitro drug release study of PVA/B-Cly self-healing hydrogels in phosphate-buffered saline (PBS; pH 7.4, 37 °C). The data, with *n* = 3, are displayed as the mean ± standard deviation.

The release of Cly from the self-healing hydrogel was assessed by employing dialysis membranes and PBS as the medium for release. The self-healing hydrogel releasing Cly exhibited a controlled release of Cly, as seen in Figure 6. Approximately 70% of the Cly was released within the first 3 h. Cly was fully released from F4 at the sixth hour. At the sixth hour, 81.49% and 94.8% of Cly was released from F2 and F3, respectively. The drug release kinetics conformed to the Korsmeyer–Peppas model, with an average R^2^ value of 0.9848 (Table 3).

The release of Cly from the PVA/B-Cly self-healing hydrogel is affected by the dimensions of the pores [36]. According to Palungan et al. (2024), there is a positive correlation between pore size and the amount of Cly released [37]. In other words, as the pore size increases, the amount of Cly released also increases. In their study, Sarkar et al. (2015) also discovered that the rate at which fatty acids are released is directly influenced by the size of the pores in gelatine-gelled emulsions. This indicates that there is a correlation between the diffusion of lipase towards the surface of fat droplets and the average size of the pores [38]. Furthermore, Li Wang et al. (2021) discovered that the assimilation and dispersion of antimicrobials were enhanced in gels exhibiting greater average pore sizes [39]. Therefore, F4 is a hydrogel with self-healing properties that exhibits a higher degree of Cly release and is fully released within a time frame of 6 h. The release kinetics of Cly from the self-healing hydrogel conformed to the Korsmeyer–Peppas release model. This model elucidates the process of drug release from a polymer system that is regulated by diffusion. The release mechanism adheres to Fickian diffusion, with a value of *n* = 0.41 (<0.89). The drug is released when the media solution enters the hydrogels, causing the hydrogels to swell. This swelling occurs due to a difference in concentration gradient between the inside and outside of the diffusion membrane. As a result, Cly diffuses out through the membrane to the dissolution media [40,41].

#### 2.2.9. In Vivo Wound-Healing Activity

**Figure 7:**
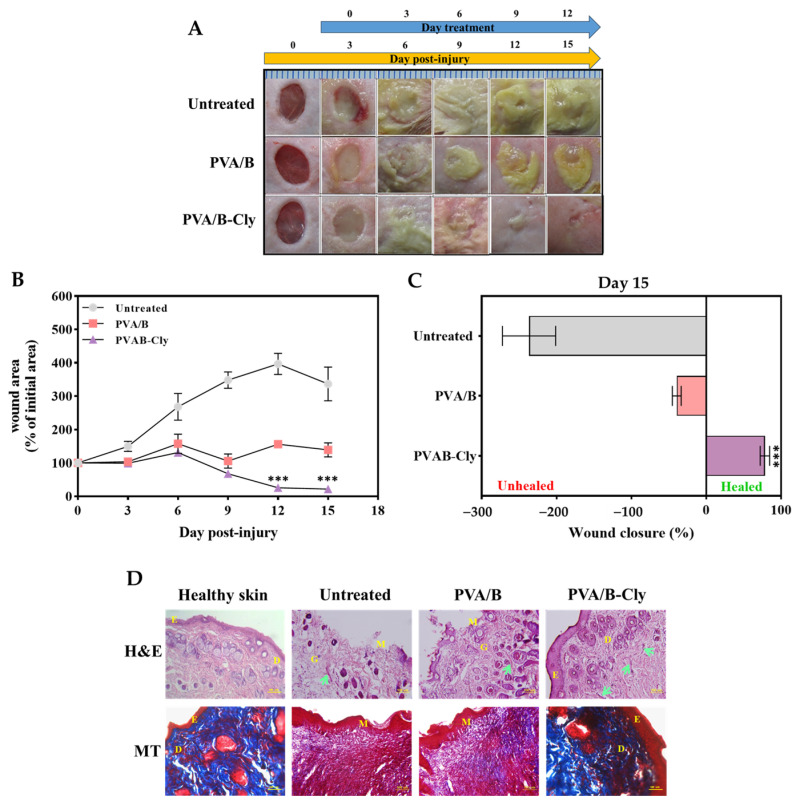
Wound-healing assay in mice. (**A**) Representative images of MRSA biofilm-infected wounds of ICR mice treated with or without PVA/B and PVA/B-Cly. (**B**) Profile showing the percentage reduction in the area of the wounds. The values represent the mean ± standard deviation (SD) of 10 separate wounds. The symbol “***” indicates that the observed difference is statistically significant (*p* < 0.05) when compared with the untreated group. (**C**) The percentage of wound closure in mice skin lesions on the last day compared with the initial 8 mm wound. (**D**) The histological examination of MRSA biofilm-infected wounds in ICR mice was conducted on day 15 using H&E and MT staining. Scale bar = 100 µm. E = epidermal, D = dermal junction, G = granulation tissue, and M = wound matrix. The green arrows represent blood vessels, specifically capillaries and neovascularization. The presence of blue hues in the MT staining images signifies the presence of collagen that is aligned in a parallel manner to the surface.

The in vivo wound-healing assay was conducted to determine if PVA/B and PVA/B-Cly self-healing hydrogels may expedite the healing process of MRSA biofilm-infected wounds (Figure 7). Visually, the application of PVA/B-Cly self-healing hydrogel treatments resulted in a decrease in the number of bacteria in the wound, leading to faster wound healing. In contrast, the untreated and blank self-healing hydrogel (PVA/B) did not have any effect on the bacterial load or size of the wound (Figure 7A). The PVA/B-Cly-treated groups exhibited a decrease in bacterial load in vivo on day 9 after the injury. The percentage of wound closure at day 15 after injury in response to PVA/B-Cly treatments was 21.50% (*p* < 0.05) (Figure 7B). The data also revealed that the wound healed 78.5% (Figure 7C). According to the presented evidence, PVA/B-Cly demonstrates efficacy in combating MRSA infection by inhibiting bacterial proliferation. In our prior investigation, we noticed that the presence of bacteria in wounds hinders the healing process by creating structures that cannot be penetrated by phagocytic cells and that are resistant to antibiotics. To prevent severe local and systemic infection and promote wound healing, it is necessary to eliminate the bacterial load on the wound bed [7]. Comparing the untreated and PVA/B groups, we discovered that the PVA/B-Cly self-healing hydrogel demonstrated a significant ability to decrease the number of bacteria and speed up wound-healing processes, such as wound closure and re-epithelialization. This effect was not observed in the untreated and PVA/B groups. Our results were in line with Chen et al. (2018) [16], who studied an injectable self-healing hydrogel that accelerates wound healing. The chitosan–konjac glucomannan had a good self-healing property, effectively inhibited bacterial growth, and promoted wound contraction and healing [42]. Another study also showed that antibacterial self-healing hydrogel promotes wound healing. The quaternized N-[3-(dimethylamino)propyl] methacrylamide copolymers self-healing hydrogel fabricated with dithiodipropionic acid dihydrazide as a cross-linking agent significantly accelerated wound healing by eradicating bacterial infection and promoting re-epithelialization in the wound model [43]. Hence, hydrogels with self-healing qualities could be valuable for promoting wound healing and protecting wounds from further damage. They can avoid secondary injuries, prolong the lifespan of dressing materials, and offer additional safeguarding to wound sites by maintaining their form through diverse self-healing mechanisms [12]. The self-healing hydrogel synthesized from PVA/borax exhibits exceptional quality, making it very effective for wound healing and making it widely utilized as a biomaterial. According to Medrano et al. (2021), injectable bone replacements composed of PVA, borax, CaCO_3_, and demineralized bone matrix show promising prospects for bone tissue regeneration. The self-healing hydrogel possesses viscoelastic properties, enabling it to flow and effectively occupy a bone deficiency [44].

The histology of H&E- and MT-stained skin specimens demonstrates the collagen deposition and skin layer morphology at day 15 post-injury. PVA/B-Cly-treated mice had healthy-looking skin, unlike the untreated and PVA/B groups (Figure 7D). The untreated and PVA/B group H&E staining showed open wounds, early epithelialization, and ulceration. In contrast, the PVA/B-Cly group samples had more fibroblast-like cells, fewer mononuclear inflammatory cells, and repaired skin that looked like healthy skin. H&E staining did not distinguish collagen fibers, whereas MT staining’s blue color did. The blue intensity on trichrome staining suggested collagen, which normally appears parallel to the surface during wound remodeling [45].

## 3. Conclusions

Our study successfully developed a self-healing hydrogel wound dressing composed of PVA/B-Cly, which has the ability to heal itself and release Cly. The formation of a sustained and regulated release of Cly is achieved by cross-linking PVA and borax, following the Korsmeyer–Peppas kinetic model with a Fickian diffusion process. Increasing the concentration of borax as a cross-linking agent results in the formation of a self-healing hydrogel that exhibits favorable physicochemical characteristics. The PVA/B-Cly self-healing hydrogel has good storage stability at a temperature of 6 ± 2 °C, with no notable decline observed in its self-healing properties. In addition, PVA/B-Cly greatly enhances the rate of wound healing and re-epithelialization in a mouse model with biofilm-infected wounds. Hence, the creation of Cly-releasing self-healing hydrogel holds great potential in improving wound healing and addressing diverse skin infections.

## 4. Materials and Methods

### 4.1. Materials

Clindamycin phosphate (Cly), polyvinyl alcohol (PVA, MW = 44.05, 87–89%), methylene blue hydrate, potassium bromide (KBr), methanol, sodium tetraborate decahydrate (borax), and phosphate buffered saline (Dulbecco A) Oxoid BR0014G were purchased from Sigma-Aldrich (St. Louis, MO, USA). The dialysis osmosis membrane with cellophane visking tubing was obtained from Medicell Membrane Ltd. (London, UK). All other reagents and solvents were of the highest analytical grade.

### 4.2. Preparation of Self-Healing Hydrogels

Self-healing hydrogels were synthesized by cross-linking PVA and borax in aqueous solution. All concentrations of PVA, borax, and Cly follow the concentrations in Table 1. For F2, PVA (4 %wt) and Cly (1 %wt) were first mixed and dissolved in water, then placed in an oven at 90 °C for 2 h until they were completely dissolved. Next, borax (0.8 %wt) was dissolved in water and heated at 90 °C until it was completely dissolved. The borax solution was added little by little to the mixed solution of PVA and Cly at a temperature of 90 °C while homogenizing (homogenizer: WiseStir^®^ HS-50A, Daihan, Republic of Korea) until a self-healing hydrogel known as PVA/B-Cly was formed. Next, self-healing was printed by pouring it into a Petri dish. A self-healing hydrogel blank without Cly (PVA/B) was prepared in the same way without the addition of Cly [14].

### 4.3. Physicochemical Characterization of the Self-Healing Hydrogels

#### 4.3.1. Organoleptic Properties

A self-healing hydrogel blank (PVA/B) and self-healing hydrogel releasing Cly (PVA/B-Cly) were observed visually, considering their shape, size, and color.

#### 4.3.2. Scanning Electron Microscopy (SEM)

The morphologies of the self-healing hydrogels PVA/B and PVA/B-Cly were observed using SEM (Jeol^®^ IT 200, Tokyo, Japan). The self-healing hydrogels resulting from freezer drying were then fixed on an aluminum plate with the help of double-sided graphite adhesive tape, then coated with a thin layer of gold that was 20 nm thick. Observations were carried out at an accelerating voltage of 10.00 Kv [46].

#### 4.3.3. Fourier Transform Infrared Spectroscopy (FTIR) Characterization

The spectra of PVA/B and PVA/B-Cly self-healing hydrogels were observed using FTIR (Shimadzu^®^ IRPrestige-21, Tokyo, Japan). The self-healing hydrogels were dried and compressed into pellets using KBr. The spectra were recorded at a resolution of 4 cm^−1^ in the wavenumber range of 4000–400 cm^−1^ [47].

#### 4.3.4. Self-Healing Behavior of PVA/B and PVA/B-Cly

PVA/B and PVA/B-Cly self-healing hydrogels were cut into two parts; one part was stained with methylene blue, and the other part was not colored. Then, the two pieces were placed together in one container and brought close together. The time needed for the self-healing hydrogel to reintegrate was recorded. This process was carried out at room temperature [15,48].

#### 4.3.5. pH of Self-Healing Hydrogel

The pH of PVA/B and PVA/B-Cly self-healing hydrogels was measured using a pH meter (EZDO^®^ PL-700 Series Bench Top pH Meter, Taipei, Taiwan). A 1 g sample of self-healing hydrogel was dissolved in 10 mL of distilled water and stirred using a magnetic stirrer until dissolved. Next, the pH of the solution was measured using a pH meter until the value displayed on the instrument was constant [24].

#### 4.3.6. PVA/B-Cly Self-Healing Drug Content

The Cly content in the PVA/B-Cly self-healing hydrogel was measured. A total of 10 mg of self-healing hydrogel was added with 10 mL of methanol to obtain a concentration of 1000 ppm. Next, it was homogenized using a magnetic stirrer (IKA^®^ C-MAG HS 7 digital, Staufen, Germany) for 30 min, sonicated (Krisbow^®^ CD 3800A Digital, Jakarta, Indonesia) for 30 min, and centrifuged (Oregon^®^ LC-04S, Zhangjiagang, China) at a speed of 3500 rpm for 15 min. A total of 500 µL of the solution was sampled then put into a 1.5 mL Eppendorf tube and filled with PBS (pH 7.4). Then, Cly levels were measured using a UV-Vis spectrophotometry device (Dynamica^®^ XB-10, Livingston, UK) at a wavelength of 211.8 nm [24].

#### 4.3.7. Swelling Ratio of Self-Healing Hydrogel

The swelling ratios of PVA/B and PVA/B-Cly self-healing hydrogels were measured by weighing the self-healing hydrogels and recording the weight as the initial weight. Next, the self-healing hydrogels were soaked in 4 mL of PBS (pH 7.4). At certain time intervals, the PBS was removed with the help of filter paper, and the weight of the self-healing hydrogels were weighed as the final weight. The swelling ratio percentage is calculated using the equation: [3].

$$\mathrm{Swelling}\;\mathrm{ratio}\;=\frac{\mathrm{Final}\;\mathrm{Weight}\;-\mathrm{Initial}\;\mathrm{weight}}{\mathrm{Initial}\;\mathrm{weight}}\times 100\%$$

#### 4.3.8. Stability Study

The stabilities of the self-healing hydrogels was tested for 28 days at three different temperature conditions. Freezer temperature (−20 °C), refrigerator temperature (6 °C), and room temperature (25 °C). At 0, 7, 14, and 28 days, changes were organoleptically observed in the self-healing hydrogel in terms of pH, self-healing time, and Cly content in the PVA/B-Cly self-healing hydrogel, as described in the previous section [49].

### 4.4. In Vitro Drug Release

Cly release from the PVA/B-Cly self-healing hydrogel was tested using a dialysis membrane. The release medium used 100 mL of PBS (pH 7.4) in a Duran bottle. The self-healing hydrogel was dissolved in PBS (pH 7.4) and added to the release medium. The temperature was set at 37 °C and was accompanied by orbital shaking at a speed of 100 rpm. At the specified time interval, 1 mL of sample was taken and immediately replaced with fresh media with the same volume. Drug levels were then measured using a UV-Vis spectrophotometer (Dynamica^®^ XB-10, Livingston, UK) at a wavelength of 211.8 nm. Drug release data were calculated cumulatively, and a release kinetic model was determined using DD Solver 1.0 program. The release kinetic model was adapted to various release kinetic models such as zero-order, first-order, Higuchi, Hixson–Crowel, and Korsmeyer–Peppas models [50].

### 4.5. In Vivo Wound Healing

The animal experiments conducted in this study adhered to the regulations outlined in document PNU-2020-2839, which align with the Korean legislation on animal studies. The Ethical Scientific Committee of Pusan National University, located in Busan, South Korea, granted approval for these experiments on 22 December 2020. We employed male ICR mice (7–8 weeks of age, Samtako Bio Korea, Osan-si, Republic of Korea). The mice were intraperitoneally anesthetized using avertin. In order to create full-thickness wounds, the hair on the back was trimmed using an electric razor, and then an 8 mm biopsy punch was used to remove the skin. Next, a suspension containing 1.0 × 108 MRSA was introduced in order to cause a biofilm infection. The self-healing hydrogel was applied topically starting from day 3 after the damage. Tegaderm^®^ and sterile gauze were employed to enclose each wound. As a control, untreated mice were used. The gauze was changed every 72 h. The wounds’ photographs were examined using ImageJ software (Version 1.52i, National Institutes of Health, Bethesda, MA, USA, 2018) in order to determine the reduction in wound size. This was performed by applying the following equation:

(1) $$\mathrm{Wound}\;\mathrm{size}\;\mathrm{reduction}\;\left(\%\right)=\frac{W_{t}}{W_{0}}\times 100$$

where *W_t_* is the area of the wound at time *t* and *W*_0_ is the area of the wound at starting time 0.

### 4.6. Statistical Analysis

The data obtained were processed in graphic form using GraphPad Prism 8.0. The statistical analysis used was IBM SPSS. If the data were normally distributed, then the one-way ANOVA analysis was carried out, whereas if the data were not normally distributed, then the Kruskal–Wallis test was carried out. The analysis results were declared significantly different if the *p* value < 0.05. Data are presented as means ± SD.

## Figures and Tables

**Table 1:** Formulation of self-healing hydrogel PVA/B and PVA/B-Cly.

Formulation Code	PVA (%wt)	Borax (B, %wt)	Clindamycin Phosphate (Cly, %wt)
F1	4	1.6	-
F2	4	0.8	1
F3	4	1.2	1
F4	4	1.6	1

**Table 2:** Healing time, pH, and drug loading of self-healing hydrogels.

Formulation Code	Healing Time (min)	pH	Drug Loading (%)
F1	14.00	7.36	NA
F2	6.96	7.56	98.72
F3	9.18	7.65	97.77
F4	12.42	7.63	98.34

**Table 3:** The release kinetics model-fitting parameters of the PVA/B-Cly self-healing hydrogels.

Kinetic Model (R^2^)					
	Zero–Order	First–Order	Higuchi	Korsmeyer–Peppas	Hixson–Crowel
F2	0.6693	0.9224	0.9738	0.9722	0.8636
F3	0.2929	0.9015	0.9545	0.9848	0.8359
F4	0.2719	0.9293	0.9485	0.9828	0.8774

## Data Availability

The original contributions presented in the study are included in the article, further inquiries can be directed to the corresponding author/s.

## References

[1] Al Fatease A., Abourehab M.A., Alqahtani A.M., Chidambaram K., Qureshi A.A., Venkatesan K., Alshahrani S.M., Abdelkader H. (2022). Polymeric/Dextran Wafer Dressings as Promising Long-Acting Delivery Systems for Curcumin Topical Delivery and Enhancing Wound Healing in Male Wistar Albino Rats. Pharmaceuticals.

[2] Alghamdi B.A., Al-Johani I., Al-Shamrani J.M., Alshamrani H.M., Al-Otaibi B.G., Almazmomi K., Yusof N.Y. (2023). Antimicrobial resistance in methicillin-resistant Staphylococcus aureus. Saudi J. Biol. Sci..

[3] Hasan N., Cao J., Lee J., Kim H., Yoo J.-W. (2021). Development of clindamycin-loaded alginate/pectin/hyaluronic acid composite hydrogel film for the treatment of MRSA-infected wounds. J. Pharm. Investig..

[4] Choi M., Hasan N., Cao J., Lee J., Hlaing S.P., Yoo J.W. (2020). Chitosan-based nitric oxide-releasing dressing for anti-biofilm and in vivo healing activities in MRSA biofilm-infected wounds. Int. J. Biol. Macromolecules.

[5] Hasan N., Cao J., Lee J., Naeem M., Hlaing S.P., Kim J., Jung Y., Lee B.-L., Yoo J.-W. (2019). PEI/NONOates-doped PLGA nanoparticles for eradicating methicillin-resistant Staphylococcus aureus biofilm in diabetic wounds via binding to the biofilm matrix. Mater. Sci. Eng. C.

[6] Chaiwarit T., Rachtanapun P., Kantrong N., Jantrawut P. (2020). Preparation of clindamycin hydrochloride loaded de-esterified low-methoxyl mango peel pectin film used as a topical drug delivery system. Polymers.

[7] Hasan N., Cao J., Lee J., Hlaing S.P., Oshi M.A., Naeem M., Ki M.-H., Lee B.L., Jung Y., Yoo J.-W. (2019). Bacteria-targeted clindamycin loaded polymeric nanoparticles: Effect of surface charge on nanoparticle adhesion to MRSA, antibacterial activity, and wound healing. Pharmaceutics.

[8] Machowska A., Klara J., Ledwójcik G., Wójcik K., Dulińska-Litewka J., Karewicz A. (2022). Clindamycin-loaded halloysite nanotubes as the antibacterial component of composite hydrogel for bone repair. Polymers.

[9] Frei C.R., Miller M.L., Lewis J.S., Lawson K.A., Hunter J.M., Oramasionwu C.U., Talbert R.L. (2010). Trimethoprim-sulfamethoxazole or clindamycin for community-associated MRSA (CA-MRSA) skin infections. J. Am. Board Fam. Med..

[10] Sadeghi S., Nourmohammadi J., Ghaee A., Soleimani N. (2020). Carboxymethyl cellulose-human hair keratin hydrogel with controlled clindamycin release as antibacterial wound dressing. Int. J. Biol. Macromol..

[11] Rani Raju N., Silina E., Stupin V., Manturova N., Chidambaram S.B., Achar R.R. (2022). Multifunctional and Smart Wound Dressings—A Review on Recent Research Advancements in Skin Regenerative Medicine. Pharmaceutics.

[12] Devi VK A., Shyam R., Palaniappan A., Jaiswal A.K., Oh T.-H., Nathanael A.J. (2021). Self-healing hydrogels: Preparation, mechanism and advancement in biomedical applications. Polymers.

[13] Tu L., Fan Y., Deng Y., Hu L., Sun H., Zheng B., Lu D., Guo C., Zhou L. (2023). Production and Anti-Inflammatory Performance of PVA Hydrogels Loaded with Curcumin Encapsulated in Octenyl Succinic Anhydride Modified Schizophyllan as Wound Dressings. Molecules.

[14] Wang Y., Shi Y., Gu Y., Xue P., Xu X. (2021). Self-healing and highly stretchable hydrogel for interfacial compatible flexible paper-based micro-supercapacitor. Materials.

[15] Al-Emam E., Soenen H., Caen J., Janssens K. (2020). Characterization of polyvinyl alcohol-borax/agarose (PVA-B/AG) double network hydrogel utilized for the cleaning of works of art. Herit. Sci..

[16] Chen Y.-N., Jiao C., Zhao Y., Zhang J., Wang H. (2018). Self-assembled polyvinyl alcohol–tannic acid hydrogels with diverse microstructures and good mechanical properties. ACS Omega.

[17] Cho S., Hwang S.Y., Oh D.X., Park J. (2021). Recent progress in self-healing polymers and hydrogels based on reversible dynamic B–O bonds: Boronic/boronate esters, borax, and benzoxaborole. J. Mater. Chem. A.

[18] Dwynda I., Zainul R. (2018). Boric Acid (H3(BO3): Recognize The Molecular Interactions in Solutions. https://osf.io/preprints/inarxiv/6wead.

[19] Abouzeid R., Shayan M., Wu T., Gwon J., Karki T.A., Wu Q. (2023). Highly flexible, self-bonding, self-healing, and conductive soft pressure sensors based on dicarboxylic cellulose nanofiber hydrogels. ACS Appl. Polym. Mater..

[20] Dave H.K., Nath K. (2018). Synthesis, Characterization and Application of Disodium Tetraborate Cross-Linked Polyvinyl Alcohol Membranes for Pervaporation Dehydration of Ethylene Glycol. Acta Chim. Slov..

[21] Karvinen J., Kellomäki M. (2022). Characterization of self-healing hydrogels for biomedical applications. Eur. Polym. J..

[22] Spoljaric S., Salminen A., Luong N.D., Seppälä J. (2014). Stable, self-healing hydrogels from nanofibrillated cellulose, poly (vinyl alcohol) and borax via reversible crosslinking. Eur. Polym. J..

[23] Huang M., Hou Y., Li Y., Wang D., Zhang L. (2017). High performances of dual network PVA hydrogel modified by PVP using borax as the structure-forming accelerator. Des. Monomers Polym..

[24] Ranjan P., Jain V., Shende S., Jain P.K. (2019). Formulation development and evaluation of emulgel of clindamycin phosphate for effective treatment of acne. J. Drug Deliv. Ther..

[25] Sringam J., Pankongadisak P., Trongsatitkul T., Suppakarn N. (2022). Improving mechanical properties of starch-based hydrogels using double network strategy. Polymers.

[26] Jones E.M., Cochrane C.A., Percival S.L. (2015). The effect of pH on the extracellular matrix and biofilms. Adv. Wound Care.

[27] Bennison L., Miller C., Summers R., Minnis A., Sussman G., McGuiness W. (2017). The pH of wounds during healing and infection: A descriptive literature review. Wound Pract. Res. J. Aust. Wound Manag. Assoc..

[28] Goswami A.G., Basu S., Banerjee T., Shukla V.K.J. (2023). Biofilm and wound healing: From bench to bedside. Eur. J. Med. Res..

[29] Derwin R., Patton D., Avsar P., Strapp H., Moore Z. (2022). The impact of topical agents and dressing on pH and temperature on wound healing: A systematic, narrative review. Int. Wound J..

[30] Ganji F., Vasheghani F.S., Vasheghani F.E. (2010). Theoretical Description of Hydrogel Swelling: A Review. Iran. Polym. J..

[31] Chang K.-Y., Chou Y.-N., Chen W.-Y., Chen C.-Y., Lin H.-R. (2022). Mussel-inspired adhesive and self-healing hydrogel as an injectable wound dressing. Polymers.

[32] Narayan S., Choudhary M. (2017). A review on stability studies of pharmaceutical products. Int. J. Appl. Pharm. Biol. Res..

[33] Kostrzębska A., Pączek K., Weselak A., Musiał W. (2023). Effect of hydrogel substrate components on the stability of tetracycline hydrochloride and swelling activity against model skin sebum. Int. J. Mol. Sci..

[34] Larrea-Wachtendorff D., Del Grosso V., Ferrari G. (2022). Evaluation of the physical stability of starch-based hydrogels produced by high-pressure processing (HPP). Gels.

[35] Abdella S., Abid F., Youssef S.H., Kim S., Afinjuomo F., Malinga C., Song Y., Garg S. (2023). pH and its applications in targeted drug delivery. Drug Discov. Today.

[36] Ghasemiyeh P., Mohammadi-Samani S. (2019). Hydrogels as drug delivery systems; pros and cons. Trends Pharm. Sci..

[37] Palungan J., Luthfiyah W., Mustopa A.Z., Nurfatwa M., Rahman L., Yulianty R., Wathoni N., Yoo J.-W., Hasan N. (2024). The Formulation and Characterization of Wound Dressing Releasing S-Nitrosoglutathione from Polyvinyl Alcohol/Borax Reinforced Carboxymethyl Chitosan Self-Healing Hydrogel. Pharmaceutics.

[38] Sarkar A., Juan J.-M., Kolodziejczyk E., Acquistapace S., Donato-Capel L., Wooster T.J. (2015). Impact of protein gel porosity on the digestion of lipid emulsions. J. Agric. Food Chem..

[39] Wang L., Fogliano V., Heising J., Dekker M. (2021). The effect of pore size on the diffusion of volatile antimicrobials is a key factor to preserve gelled foods. Food Chem..

[40] Grassi M., Grassi G., Lapasin R., Colombo I. (2006). Understanding Drug Release and Absorption Mechanisms: A Physical and Mathematical Approach.

[41] Wen L., Liang Y., Lin Z., Xie D., Zheng Z., Xu C., Lin B. (2021). Design of multifunctional food packaging films based on carboxymethyl chitosan/polyvinyl alcohol crosslinked network by using citric acid as crosslinker. Polymer.

[42] Chen H., Cheng J., Ran L., Yu K., Lu B., Lan G., Dai F., Lu F.J.C.p. (2018). An injectable self-healing hydrogel with adhesive and antibacterial properties effectively promotes wound healing. Carbohydr. Polym..

[43] Bo Y., Zhang L., Wang Z., Shen J., Zhou Z., Yang Y., Wang Y., Qin J., He Y. (2021). Antibacterial hydrogel with self-healing property for wound-healing applications. ACS Biomater. Sci. Eng..

[44] Medrano-David D., Lopera A.M., Londoño M.E., Araque-Marín P. (2021). Formulation and characterization of a new injectable bone substitute composed PVA/borax/CaCO3 and Demineralized Bone Matrix. J. Funct. Biomater..

[45] Hasan N., Lee J., Ahn H.-J., Hwang W.R., Bahar M.A., Habibie H., Amir M.N., Lallo S., Son H.-J., Yoo J.-W.J.P. (2021). Nitric oxide-releasing bacterial cellulose/chitosan crosslinked hydrogels for the treatment of polymicrobial wound infections. Pharmaceutics.

[46] Gonçalves R.C., Signini R., Rosa L.M., Dias Y.S.P., Vinaud M.C., Lino R.d.S. (2021). Carboxymethyl chitosan hydrogel formulations enhance the healing process in experimental partial-thickness (second-degree) burn wound healing. Acta Cir. Bras..

[47] Luo J., Shi X., Li L., Tan Z., Feng F., Li J., Pang M., Wang X., He L. (2021). An injectable and self-healing hydrogel with controlled release of curcumin to repair spinal cord injury. Bioact. Mater..

[48] Weerawan N., Chalitangkoon J., Monvisade P. (2022). Self-healing hydrogels based on sodium carboxymethyl cellulose/poly (vinyl alcohol) reinforced with montmorillonite. Biointerface Res. Appl. Chem.

[49] Kim J.O., Noh J.-K., Thapa R.K., Hasan N., Choi M., Kim J.H., Lee J.-H., Ku S.K., Yoo J.-W. (2015). Nitric oxide-releasing chitosan film for enhanced antibacterial and in vivo wound-healing efficacy. Int. J. Biol. Macromol..

[50] Shafiei F., Ghavami-Lahiji M., Kashi T.S.J., Najafi F. (2021). Drug release kinetics and biological properties of a novel local drug carrier system. Dent. Res. J..

